# Comparing the disk-diffusion and agar dilution tests for *Neisseria gonorrhoeae* antimicrobial susceptibility testing

**DOI:** 10.1186/s13756-016-0148-x

**Published:** 2016-11-24

**Authors:** Hsi Liu, Thomas H. Taylor, Kevin Pettus, Steve Johnson, John R. Papp, David Trees

**Affiliations:** 1 Division of STD Prevention, NCHHSTP, Atlanta, USA; 2 Division of Laboratory Systems, CSELS, Centers for Disease Control and Prevention, 1600 Clifton Rd, Atlanta, GA 30333 USA

**Keywords:** Antibiotic, Susceptibility testing, Ceftriaxone, Cefixime, Neisseria, Gonorrhoeae

## Abstract

**Background:**

We assessed the validity of testing for antimicrobial susceptibility of clinical and mutant *Neisseria gonorrhoeae* (GC) isolates by disk diffusion in comparison to agar dilution, and Etest® (bioMerieux, France), respectively, for three third generation extended spectrum cephalosporins (ESC): ceftriaxone (CRO), cefixime (CFX), and cefpodoxime (CPD).

**Methods:**

One hundred and five clinical isolates and ten laboratory-mutants were tested following Clinical Laboratory Standard Institute (CLSI) and manufacturer’s standards for each of the three methods. The measured diameters by the disk diffusion method were tested for correlation with the MIC values by agar dilution. In addition, comparisons with the Etest® were made. Categorical results for concordance, based on standard CLSI cutoffs, between the disk diffusion and the other two methods, respectively, were tested using the Chi-square statistics. Reproducibility was tested for CFX across a 6-month interval by repeated disk tests.

**Results:**

Across all 115 specimens, the disk diffusion tests produced good categorical agreements, exhibiting concordance of 93.1%, 92.1%, and 90.4% with agar dilution and 93.0%, 92.1%, and 90.4% with Etest®, for CRO, CFX, and CPD, respectively. Pearson correlations between disk-diffusion diameters and agar dilution MIC’s were -0.59, -0.67, and -0.81 for CRO, CFX, and CPD, respectively. The correlations between disk diffusion and Etest® were -0.58, -0.73, and -0.49. Pearson correlation between the CFX disk readings over a 6-month interval was 91%.

**Conclusions:**

Disk diffusion tests remain to be a useful, reliable and fast screening method for qualitative antimicrobial susceptibility testing for ceftriaxone, cefixime, and cefpodoxime.

## Background

*Neisseria gonorrhoeae* (*N. gonorrhoeae*) is one of the most common sexually transmitted pathogens. Its high morbidity and associated medical and socio-economic costs make it one of the major public health issues in the U.S. and in the world. Approximately 300,000 cases are reported to the CDC each year [1]. Because many cases could be asymptomatic, a recent estimate indicates that there may be more than 820,000 infected individuals in the U.S [2]. Globally, many areas are experiencing significant rise in reported cases of gonorrhea. According to the World Health Organization (WHO), in 2008 there were 106 million new cases of gonorrhea worldwide [3].

CDC currently recommends that for uncomplicated genital, rectal, and pharyngeal gonorrhea, a combination of two drugs should be used; specifically, ceftriaxone 250 mg is administered intramuscularly (IM) in a single dose, plus azithromycin 1 g orally in a single dose. When ceftriaxone is not available, cefixime 400 mg orally in a single dose is recommended to replace ceftriaxone in combination with 1 g oral azithromycin [4, 5]. Treatment guidelines also recommend individuals with pharyngeal infections return for medical consultation 1 week post therapy to ensure success of therapy.

It is not clear whether combining two antimicrobials with different mechanisms of action (e.g., ceftriaxone plus azithromycin) can delay the rise of isolates that are resistant to the extended spectrum cephalosporins (ESC) [6]. A recent report from Canada showed a significant decline in decreased susceptibility to cephalosporins but at the same time increased azithromycin resistance [7].

Despite the use of dual drug therapy, both CDC and the WHO have warned that gonorrhea will soon become more difficult to treat. This is because gonococcus is well known for its ability to develop antimicrobial resistance against first-line therapies within a short duration of a drug’s introduction [8–11].

Until new drugs for treating gonorrhea are discovered, there is urgent need for public health professionals to closely monitor antimicrobial susceptibility of gonococci. Quick identification of isolates that potentially possess reduced susceptibility to cephalosporins is critical to controlling the spread of drug-resistant gonococcal organisms. The recent emergence of isolates with reduced antibiotic susceptibility across the country has further alerted physicians and epidemiologists to the urgent need to closely monitor such activities [10].

Three common tests are routinely used by laboratories to determine the antimicrobial susceptibility of *N. gonorrhoeae*. The agar dilution test is the gold standard and is used mostly by reference laboratories. We reported recently that the Etest® (bioMerieux, France) is suitable to serve as an alternative test [12]. Both agar dilution and Etest methods report an MIC value which can be easily used by physicians or epidemiologists to determine treatment options. These two tests are appropriate for finding organisms with increased MIC values. While these tests are our first choice, they are not routinely used by all laboratories.

A third test, the disk diffusion test, is more commonly used by microbiological laboratories and hospitals world-wide to determine the antimicrobial susceptibility of many organisms against wide-spectrum antibiotics [9, 12–15]. The test is simple and widely accepted by many laboratories. Its use, however, is not without limitations. Disk diffusion test results are observed as a diameter of inhibition-zone. The diameter is relatively imprecise and often cannot convert to a MIC value, but rather provides a categorical classification of susceptible, intermediate, or resistant phenotypes [10]. For the treatment of gonococci with cephalosporins, only susceptible and non-susceptible categories are accepted for classifications [13, 14, 16].

In the past 20 years, the number of laboratories using the disk diffusion method for *N. gonorrhoeae* antimicrobial susceptibility testing has declined. In 1989, 86% of public health laboratories surveyed used the disk diffusion method [17]. In a study performed in New York in 2000, approximately 37.4% of laboratories used disk diffusion [18]; in a 2012 study [19], 47% of laboratories were utilizing disk diffusion tests for *N. gonorrhoeae* antimicrobial susceptibility. Thus the disk diffusion method remains the choice of many reference and regional laboratories and hospitals to survey gonococcal drug susceptibility. However, without sufficient clinically ESC resistant isolates, it is not clear whether the disk diffusion method can reliably detect future ESC resistance.

In this report, we compared the disk diffusion method with the current gold standard agar dilution and a potential alternative Etest method. We analyzed the suitability and reliability of disk diffusion to monitor susceptibility of *N. gonorrhoeae* isolates of the most commonly used cephalosporins (ceftriaxone and cefixime). In addition, we included 10 laboratory generated mutants with raised MIC level against these common ESCs to simulate non-susceptible isolates. Cefpodoxime was also included in this study because it was used in selecting laboratory generated mutants.

## Methods

### *N. gonorrhoeae* isolates

One hundred and five confirmed *N. gonorrhoeae* isolates from various locations in the US, including Gonococcal Isolate Surveillance Project (GISP) isolates and reference strains, were used. In addition, 10 specimens of laboratory-derived mutants that are non-susceptible to cefpodoxime were selected. All 115 isolates were used in all three tests. All isolates were confirmed by passage and selection using the modified Thayer-Martin Medium (Scientific Resource Program [SRP], CDC).

### Mutation generation

The laboratory generated mutations were selected for increased cephalosporin MICs by exposing parent strains SPN284 or GC3502 to elevated concentrations of cefpodoxime (3.0 ug/ml or 4.5 ug/ml) [20]. The specific concentrations of cefpodoxime were included in enriched GC agar base medium plates and 2-4x10^11^ CFU of parent strain were inoculated. The resulting colonies were subcultured and maintained on GC agar base medium supplemented with 1% IsoVitaleX ^TM^ [Sigma-Aldrich, MO].

### Antimicrobial susceptibility testing

Agar dilution tests and disk diffusion tests (BD BBL Sensi-Disc, Becton, Dickinson and Company, MD, U.S.A.) were performed by using the GC agar base medium supplemented with 1% IsoVitaleX ^TM^. Both tests were performed according to the Clinical and Laboratory Standards Institute (CLSI) agar dilution or disk diffusion methods [21, 22]. The Etests were performed according to the manufacturer’s recommendations (bioMerieux, France). Disk diffusion tests and Etests were performed at the same time while agar dilution tests were performed by itself due to the complexity nature of the test. Tests were prepared by suspending colonies of *N. gonorrhoeae* from an overnight culture of Chocolate II agar (Scientific Resources Program, SRP, CDC) into Muller Hinton Broth (Difco Laboratories, MI) and adjusted to an optical density (BioMate3, Thermo-Fisher Scientific) equal to that of a 0.5 McFarland standard. The organisms were evenly spread on the surface of a 10x150 mm GC base agar plate using a cotton swab and allowed to dry for about 10 min before the disks were applied to the plate and duplicated plates were performed. For the agar dilution method, a dilution of the suspension approximately 10^4^ CFU per spot was inoculated within 15 min of preparation onto the GC base agar surface with a Steers inoculator. The plates were incubated at 35 °C in 5% CO_2_ for 20–24 h. The minimum inhibitory concentrations (MICs) were interpreted by reading growth inhibition (agar dilution) or the diameter of the inhibition zone measured using a ruler (disk diffusion). When replicates have different values, we report the test value which has smaller diameter or larger MIC. A panel of 7 quality control organisms: *N. gonorrhoreae* ATCC 49226, F28, P681E, CDC10328, CDC10329, SPJ15, and SPL4 were included in each assay for validation [23]. The disk diffusion and Etest methods were performed simultaneously [12]. The Agar dilution was performed according to CLIA specified guidelines.

### Statistical analyses

Results between the disk diffusion, the agar dilution, and Etest can be compared and quantified using the CLSI cutoffs and categorical levels specified. Both agar dilution and Etest report MICs (μg/ml) which represent a continuous metric of per-unit concentrations. Disk diffusion provides an inhibition zone diameter which is also a continuous metric but in mm of distance. Accordingly, statistical comparisons between the MIC concentrations and the continuous metric diameter were performed using simple correlation and linear regression (Statistical Analysis System, Version 9.4). For all three tests, there exist CLSI standard cutoff values [18] to define susceptibility and non-susceptibility of the ESC spectrum antibiotics based on whether a particular test result is above, equal to, or below the cutoff. Hence the tests can be compared as a categorical outcome: susceptible (S) or non- susceptible (nS). Simple 2x2 tables and proportions concordant or not, between the pairwise test comparisons, are provided (Table 1). The specific threshold (cutoff) values for each test and drug are included in the table. To test for reproducibility, the disk diffusion test of cefixime was repeated at multiple time points over a 6-month period.

**Table 1:** Categorical Comparisons of the Susceptibility Testing Results of Disk Diffusion with Agar Dilution

Disk diffusion, zone diameter		Agar dilution, MIC		
		Susceptible	Non-Susceptible	
Ceftriaxone (CRO)		<=0.25 ug/ml	>0.25 ug/ml	Totals
Susceptible	> = 35 mm	97 (84.4%)	0 (0.00%)	97 (84.4%)
Non- Susceptible	<35 mm	8 (7.0%)	10 (8.7%)	18 (15.6%)
	Totals	105 (91.3%)	10 (8.7%)	115 (100.0%)
Cefixime (CFX)		<=0.25 ug/ml	>0.25 ug/ml	Totals
Susceptible	> = 31 mm	94 (81.7%)	0 (0.0%)	94 (81.7%)
Non-Susceptible	<31 mm	9 (7.8%)	12 (10.4%)	21 (18.3%)
	Totals	103 (89.6%)	12 (10.4%)	115 (100.0%)
Cefpodoxime (CPD)		<=0.5 μg/ml	>0.5 μg/ml	Totals
Susceptible	> = 29 mm	58 (50.4%)	7 (6.1%)	65 (56.5%)
Non-Susceptible	<29 mm	4 (3.5%)	46 (40.0%)	50 (43.5%)
	Totals	62 (53.9%)	53 (46.1%)	115 (100.0%)

## Results

**Fig. 1:**
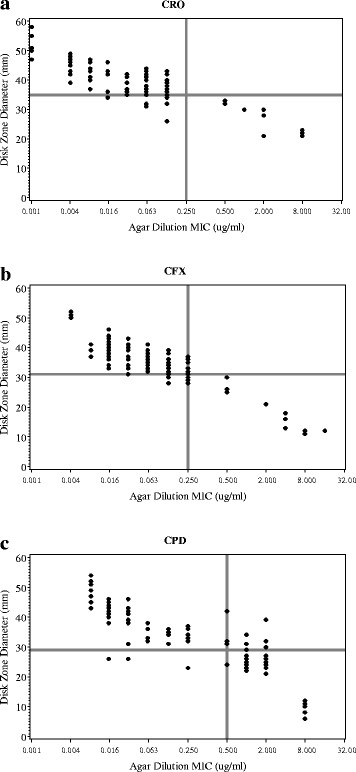
Plot of disk-diffusion zone diameter (mm) against agar-dilution minimum inhibition concentration (MIC, μg/ml), with CLSI threshold cutoffs for susceptibility. A total of 115 isolates were tested. **a**. Ceftriaxon (CRO), **b**. Cefixime (CFX), **c**. Cefpodoxime (CPD). x-axis: Agar-dilution (MIC;μg/ml). y-axis: Disk-diffusion zone diameter (mm)

The MICs measured by the agar dilution test and the diameter (mm, inhibition zone) of the disk diffusion test exhibit strong linear relationships for all three antibiotics. The Pearson’s correlations between disk diffusion and agar dilution test were -0.59, -0.67, and -0.81 (*p* < 0.0001), respectively, for ceftriaxone, cefixime, and cefpodoxime (Fig. 1a, b, c). Previously, the Etest was shown to perform comparably to the agar dilution test [12]. When disk diffusion was compared with Etest method, similar results were obtained. The correlations between disk diffusion and Etest for the three cephalosporins were -0.58, -0.73, and -0.49, respectively. These results include all 115 pairs of tests, including clinical and mutant specimens, to insure no artificial range limitations on the correlations. The disk diffusion results agreed well with both tests across the full range of current clinical and potentially less susceptible strains for all three antibiotics.

The concordance of disk diffusion to agar dilution of the categorical results were 93.0%, 92.1%, and 90.4% for ceftriaxone, cefixime, and cefpodoxime, respectively (Table 1). For disk diffusion and Etest, respective concordance rates were 93.0%, 92.1%, and 90.4%, respectively.

**Fig. 2:**
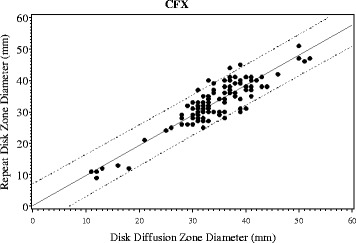
Plot of disk-diffusion zone diameter (mm) against itself at two different points in time, with fitted line and 95% confidence band, for CFX. x-axis: Disk-diffusion zone diameter, time A (mm). y-axis: Repeat Disk-diffusion zone diameter, time B (mm)

To test for reproducibility, the disk diffusion test of cefixime was repeated for each specimen. When the results of paired tests were compared, a 91% correlation (*p* < 0.0001) was observed (Fig. 2).

## Discussion

In this report, we studied the suitability and reliability of disk diffusion to monitor susceptibility of *N. gonorrhoeae* isolates of the most commonly used third generation cephalosporins (ceftriaxone and cefixime). The results and conclusions is limited to the three ESCs we tested and should not be extended without additional study to other molecules such as azithromycins or quinolones. We have demonstrated that the disk diffusion test had good correlations and categorical concordance when compared with current gold standard the agar dilution method (Fig. 1, Table 1). Disk diffusion also exhibited good reproducibility for cefixime (Fig. 2). This finding is important because the disk diffusion test remains a common method used by many regional laboratories and hospitals for detection of antimicrobial resistance, and the threat of reduced gonococcal susceptibility to extended spectrum cephalosporin may be imminent [10, 11, 24].

At present, there are few reported clinical cases of gonorrhea with isolates that demonstrate decreased ESC susceptibility [25–27]. In this study, we selected ten laboratory-generated mutants based on their significantly reduced in vitro susceptibility to three cephalosporins. These mutants also have non- susceptible phenotypes to cefixime and ceftriaxone as tested by agar dilution methods and Etest [12, 20] (Fig. 1). These high-MIC mutants (MIC values: 2–8 ug) were used to simulate non-susceptible (or clinically resistant) phenotypes and test whether disk diffusion method could reliably detect future clinical isolates that are resistant to ESCs. All ten isolates showed good categorical agreement. This observation is important because it suggests that disk diffusion remains appropriate as a routine method to detect isolates with non-susceptibility phenotype.

GC-base medium and appropriate supplements (such as IsoVitaleX) are recommended by the CLSI for testing antibiotic susceptibility of *N. gonorrhoeae* species [16, 22]. However, in some regions clinical laboratories routinely use Chocolate agar (e.g., Choc II) as testing medium. It has been reported that testing using the Chocolate agar generates results similar to those of GC base. However, a small discrepancy may result in missing isolates that fall into the potential non-susceptible category or generate false positives. Liao et al. have observed a 5.5% false resistance rate when chocolate agar is used compared with GC-base [28]. Thus, it is advisable that all laboratories using GC-base medium when performing tests of gonococcus.

According to the CLSI, the ESC antimicrobial susceptibility testing results for *N. gonorrhoeae* are classified into two categories, susceptible and non-susceptible; intermediate and resistant categories are not designated at this time [21, 22]. For cefixime and ceftriaxone, isolates with MICs greater than 0.25 ug/ml (or > = 0.5 ug/ml) are considered not susceptible to these drugs. For epidemiological purposes the CDC sometimes classifies isolates with cefixime MICs > = 0.25 ug/ml and ceftriaxone MICs > = 0.125 ug/ml as isolates having reduced-susceptibility and an increase in numbers of reduced susceptibility is a warning sign for clinicians [4, 5, 8]. This type of assessment cannot be achieved using the disk diffusion test because at this time breakpoints (in mm) are not available. Regardless, the GC-disk diffusion test appears appropriate for qualitative antibiotic susceptibility testing for clinicians to determine and choose the appropriate ESCs for treatment when no other testing alternatives are available.

However, the disk diffusion test has additional limitations that should be considered. Training and experience are required both for performing and reading the results. Further, the test is labor-intensive and has similar limitations to other culture-based tests. Reading the disk diffusion test can be subjective, human errors can affect the outcome, and despite good categorical classifications, it may have somewhat larger reproducibility variations than the Etest or agar dilution test. In this study, the same batch of disks was used throughout the experiments and the Etest strips were from one lot. Although different batches of agar plates were used, there is no reason to suspect that this variation had a major impact on results. In addition, one technician performed and read all tests. Given the nature of reading zone diameters and Etest strips, we might expect higher variability if results were read by multiple technicians.

## Conclusion

For public health laboratories performing susceptibility testing of *N. gonorrhoeae* for ceftriaxone, cefixime, and cefpodoxime, disk diffusion test remains a viable method which produces results comparable to the current gold standard, agar dilution.

## Abbreviations

CFX: Cefixime
CLSI: Clinical Laboratory Standard Institute
CPD: Cefpodoxime
CRO: Ceftriaxone
ESC: Extended spectrum cephalosporins
GC: *Neisseria gonorrhoeae*
GISP: Gonococcal Isolate Surveillance Project
IM: Intramuscularly
MIC: Minimum inhibitory concentration
NS: Non-susceptible
S: Susceptible
SRP: Scientific Resource Program
WHO: World Health Organization
